# A Context-Aware EEG Headset System for Early Detection of Driver Drowsiness

**DOI:** 10.3390/s150820873

**Published:** 2015-08-21

**Authors:** Gang Li, Wan-Young Chung

**Affiliations:** Department of Electronic Engineering, Pukyong National University, Busan 608-737, Korea; E-Mail: ligang@pknu.ac.kr

**Keywords:** driver drowsiness detection, EEG, gyroscope, slightly drowsy events, mobile application

## Abstract

Driver drowsiness is a major cause of mortality in traffic accidents worldwide. Electroencephalographic (EEG) signal, which reflects the brain activities, is more directly related to drowsiness. Thus, many Brain-Machine-Interface (BMI) systems have been proposed to detect driver drowsiness. However, detecting driver drowsiness at its early stage poses a major practical hurdle when using existing BMI systems. This study proposes a context-aware BMI system aimed to detect driver drowsiness at its early stage by enriching the EEG data with the intensity of head-movements. The proposed system is carefully designed for low-power consumption with on-chip feature extraction and low energy Bluetooth connection. Also, the proposed system is implemented using JAVA programming language as a mobile application for on-line analysis. In total, 266 datasets obtained from six subjects who participated in a one-hour monotonous driving simulation experiment were used to evaluate this system. According to a video-based reference, the proposed system obtained an overall detection accuracy of 82.71% for classifying alert and slightly drowsy events by using EEG data alone and 96.24% by using the hybrid data of head-movement and EEG. These results indicate that the combination of EEG data and head-movement contextual information constitutes a robust solution for the early detection of driver drowsiness.

## 1. Introduction

Driver drowsiness is a major cause of mortality in traffic accidents worldwide. The U.S. National Highway Traffic Safety Administration reports that drowsy driving is the cause of an estimated 40,000 injuries and 1550 deaths in car crashes every year [1]. Also, the Korean Expressway Cooperation reports that, from 2010 to 2013, 1223 people died in Korean highway traffic accidents, 31% of which could be attributed to driver drowsiness [2,3]. Many of these deaths could be avoided if driver drowsiness could be properly monitored and drivers are given early warnings.

Driver drowsiness, that is, excessive sleepiness, is more likely to happen when a person is driving for extended periods in monotonous environments, such as on a highway. The standard clinical tests for measuring sleepiness are the Multiple Sleep Latency Test (MSLT) and the Maintenance of Wakefulness Test (MWT), combined with polysomnography datasets [4]. These measurements are very expensive and cumbersome to perform (at least eight channels are needed: four EEG, two electrooculogram (EOG), one electromyogram, and one electrocardiogram (ECG) [4]); it would be practically impossible to use these methods to detect driver drowsiness in an actual driving environment. For instance, the use of multiple sensors would be uncomfortable for the driver and could even impede his or her movement. Thus, there is a strong demand for an easy-to-use driver drowsiness detection (DDD) system.

To enable the detection of driver drowsiness both simply and inexpensively, many methods have been proposed, including vehicle-based methods (such as the lane departure warning system [5] and the steering wheel movement system [6,7,8]), video-based methods (such as the detector of the degree (percentage) of eyelid closure over the pupils over time [9,10,11,12]), and physiological-signal-based methods (such as those based on the ratio of low frequency to high frequency of heart rate variability [13,14] and EEG (brain waves) [15]). Among these methods, physiological-signal-based methods are considered to be the most reliable means of detection as these signals provide an indication of the true internal state of the driver [16]; and compared to other physiological signals, the EEG, that is a non-invasive physiological means of measuring brain activity, is considered to have the closest relationship with drowsiness [4,17,18,19]. Over the years, Lin’s group is committed to detect driver drowsiness using EEG alone. For example, in 2010, his group developed a real-time DDD system, which consists of a wireless EEG headband and a self-developed portable device, which is embedded with a binary threshold-based decision-making model [20]. In November 2012, his group developed an EEG headset and Android smartphone-based driver drowsiness monitoring and management system [21]. In more recent work [22], his group further proposed a driver vigilance monitoring system, which is based on a wireless EEG headband and a support vector regression model enabled Android tablet device.

These systems, which integrate portable smart devices and EEG together, might lead to low-cost and simple-to-use DDD solutions. However, for practical purpose, a possible drawback is that the inevitable head movements caused by yawning, rubbing face or eyes and moving restlessly on chair when drivers are slightly drowsy [23], would significantly influence the EEG signal quality and result in unreliable detection result. Therefore, using EEG alone is not robust to recognize a drowsy driver’s early features, when feedback might be the most effective. If taking EEG as the main signals for DDD, then the information of head movements can be regarded as contextual information that could be easily captured by motion sensor. For example, Vural *et al.* [24] proposed to use accelerometer to measure the head movements. They mentioned that head motion increased as the driver became drowsy and the head would become still just before falling asleep. One limitation of this study is that the head motion was measured using only one dimension of the accelerometer. Regan *et al.* [25,26] proposed to use gyroscope to measure head movements. Based on a commercial device with integrated gyroscope [27], they successfully recognized the head-movement-related artifacts in EEG signals. Nevertheless, the head movements mentioned were intentioned movements that were instructed by researchers instead of the natural movements occurred in a real-life application circumstances. Also, the usage of two-axis (*X* and *Y* axis) gyroscope limits the system to a low-directional-resolution output. Compass, accelerometer and gyroscope are commonly-used motion sensors. Among the three sensors, compass and accelerometer sense magnetic north and gravity as the external references respectively. However, gyroscope is very different. It senses its own rotation without any external reference needed. So, whenever and wherever the head stopped, its sensory value goes to zero, which is very suitable to detect the intensity of head movements.

This study aims to enrich the EEG data with the intensity of head-movement by integrating three-axis gyroscope sensor into the EEG headset. We would like to determine if using a combination of EEG and head-movement parameters would increase the ability to earlier predict driver drowsiness compared to using EEG signals alone. To achieve this goal, this study includes the following aspects: (1) design and implement a low-power, wireless and context-aware EEG acquisition headset with integrated feature extraction processor; (2) design and implement a mobile Data-to-Knowledge platform using smartphone; (3) test the Data-to-Knowledge platform by driving simulation experiment. We reviewed studies about EEG-based DDD in the past decade from well-known literature pools including IEEE Xplore, ScienceDirect, and SpringerLink. To the author’s best knowledge, this is the first study attempting to detect driver drowsiness using EEG signals enriched with head-movement context information. Thus, the contributions of this work are twofold: (1) the full design for a wireless context-aware EEG system is described; (2) the concept of early detection of driver drowsiness using context-aware EEG is confirmed.

The rest of the paper is organized as follows. Section 2 presents the system design. Section 3 presents the system evaluation design for simulated driving. Section 4 presents the evaluation results. Further discussion about the evaluation results is presented in Section 5. Section 6 concludes the paper with future work.

## 2. System Design

**Figure 1:**
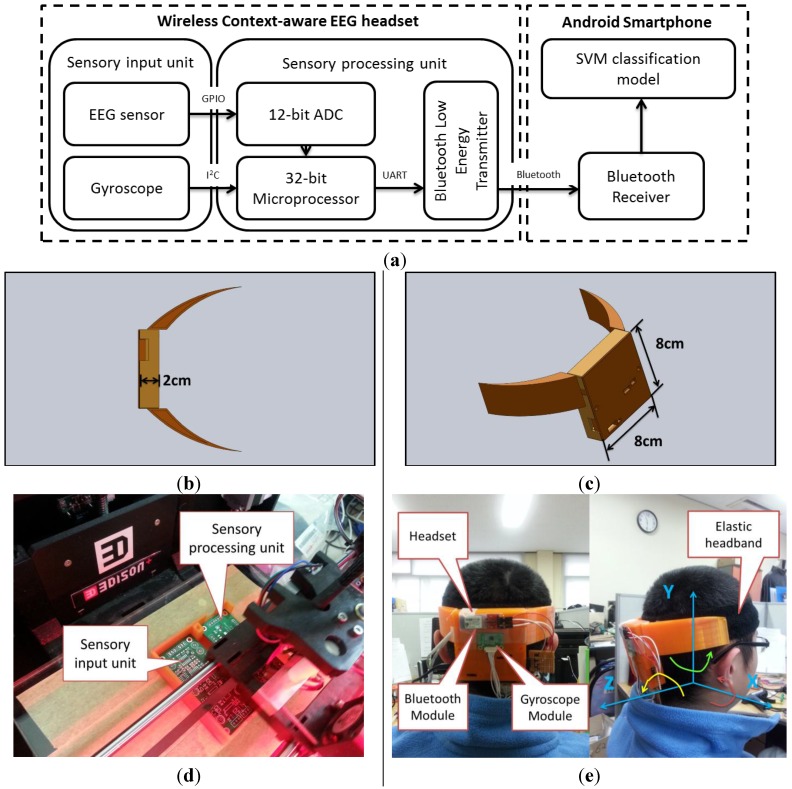
(**a**) Block diagram of the proposed system; (**b**) The top view of the designed headset; (**c**) The full view of the headset; (**d**) The fabricating procedure using 3D printer; (**e**) The fabricated headset prototype.

Figure 1 shows the proposed DDD system, which consists of a wireless context-aware EEG headset and a smartphone (Android 4.4.2, Samsung, Suwon, South Korea,). The EEG headset is powered by a 3.6 V 2600-mAh lithium-ion battery and incorporates a sensory input unit (SIU) as well as a sensory processing unit (SPU). The analog data from the SIU are converted to digital data by the SPU’s built-in 12-bit analog-to-digital converter (ADC) and stored in the 20 K static random access memory. Then, the digital data are transformed by Fast Fourier Transformation (FFT) algorithm directly inside of the 32-bit processor on the SPU. Then, the extracted FFT-based EEG and head movement features are wirelessly transmitted to the smartphone via a Bluetooth Low-Energy (BLE) module [28]. The smartphone puts these features into a support vector machine (SVM)-based classification model to automatically estimate the driver drowsiness level. The following sections introduce in detail the major components of this system.

### 2.1. Wireless Context-Aware EEG Headset

Traditional wired EEG acquisition systems were commonly seen in EEG-based DDD studies, such as [15,18,29,30,31]. These systems could be acceptable for research purposes, but essentially impossible for practical use due to being so cumbersome. This limitation can be overcome by wireless EEG acquisition systems, which eliminate the wire connection between the EEG sensing part and the Data-to-Knowledge part, using wireless transmission technology such as Bluetooth, Zigbee, or some other proprietary radio frequency unit. In this case, wireless EEG acquisition systems can be manufactured easily and offer several desirable advantages, such as small size and light weight. These desirable aspects of wireless EEG acquisition systems make them very suitable for real-life DDD applications. In this study, the developed wireless context-aware EEG headset consists of two parts: SIU and Bluetooth-enabled SPU. As can be seen in Figure 1, for wearable purposes, the two units are put into a specifically designed case which is fabricated by 3D printer, which is then connected to an elastic headband via two snap buttons.

#### 2.1.1. SIU

**Figure 2:**
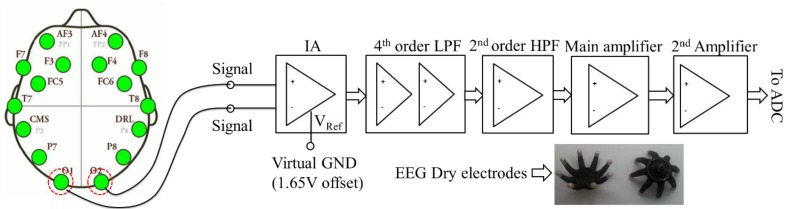
Structure of EEG bio-potentials conditioning circuit and a pair of commercial dry electrodes used. The locations marked by red dotted circles are the locations EEG electrodes attached in this study.

The SIU consists of three EEG dry electrodes, an EEG bio-potentials conditioning circuit and a three-axis gyroscope module. Among the three dry electrodes, one ear-clip electrode, from Laxtha Co., Ltd [32], serves as a ground electrode which is placed on the earlobe and given 1.65 V bias to satisfy the required condition of the SPU’s ADC which is powered by a single positive reference voltage (+3.3 V). Two dry electrodes, from Cognionics Co., Ltd [33], are used as EEG signal electrodes. They are specifically designed for hairy regions. Thus, in this study, they are placed at occipital regions O1 and O2, where is highly correlated with the driver’s vigilance level [34]. Before applying them to EEG bio-potentials conditioning circuit (as shown in Figure 2), the driver circuit for the dry electrodes which is adapted from [35] is implemented. The comparison between conventional wet Ag/AgCl electrodes and the dry electrodes can be found in Section 4.

EEG signals are usually weak and easy to be interfered by undesired noises (EEG signals typically have amplitude in the range of 10 µ–100 µV). Therefore, both amplifying and filtering are required for further signal processing. In addition, human skin typically provides source impedance on the order of 1 M–5 MΩ. Thus, amplifiers must match the source impedance or have greater input impedance than the source skin impedance to acquire EEG signals. For these reasons, the output of the bipolar electrode is transferred to EEG bio-potentials conditioning circuit and firstly amplified by an instrument amplifier (IA: INA28, Texas Instruments) with 16.5 × gains. The INA128 is a low-power Rain-to-Rain IA which features a high differential input-impedance of 10GΩ || 2pF and a high common mode rejection ratio of 100 dB when the gains are 10×. Therefore, INA128 can balance the electrode-skin impedance well and reject common mode signals as much as possible. Additionally, in order to acquire the most useful EEG bands for DDD application (θ (4–7 Hz), α (8–13 Hz) and β (13–30 Hz)) [12,13,19,20,21,22,36], the cutoff frequencies of the Low Pass Filter (LPF) and High Pass Filter (HPF) are set to 4 Hz and 30 Hz, respectively. The output signal of IA is transferred to 2nd-order Butterworth HPF and then filtered by the 4th-order Butterworth LPF. Finally, the signal is amplified by the main and 2nd non-inverting amplifier with 151 × and 12 × gains. The significance of using non-inverting amplifier is that it features high input impedance and low output impedance, which is similar to voltage follower. The amplifier used here is LMC6464 from Texas Instruments, which is a low-power operation amplifier with Rail-to-Rail input and output. A notch filter for power-line noise was not included in this circuit because there are no main power outlets in the car environment. For indoor tasks, such as the driving simulation experiment, a digital notch filter was directly implemented on chip to remove 60 Hz power-line noise in real-time.

We use L3G4200D as the sensor platform for collecting the gyroscope data. The L3G4200D is from ST semiconductor, which is a low-power three-axis digital output gyroscope. It integrates LPF, HPF and 16-bit ADC together and also supports I^2^C and SPI digital output interface. In this study, the L3G4200D are attached on EEG headset using expansion board. The 16-bit sensor readings are transferred directly to the SPU via I^2^C connection. As shown in Figure 1e, the location of the gyroscope is around the center part of the squared case of the headset. The directions of the three axes are also shown in Figure 1e, where *X*, *Y*, *Z* axes direct to the right-left, up-down and front-rear directions, respectively. Therefore, the head-movements around the three axes can be captured. For example, *X* axis is used to capture yield and look up head-movements, like yawning. *Y* axis is used to capture left-right head shaking movements. *Z* axis is used to capture left-right head swaying movements.

#### 2.1.2. SPU

The SPU consists of a self-developed micro-controller unit (MCU) system and a commercial BLE transmitter. The MCU is STM32F103CB from ST semiconductor. It operates at 3.3 V with a clock speed of 8 MHz. The MCU’s state-machine loop repeats at an interval equal to the sampling rate of 128 Hz. STM32 MCU was chosen because it is 32-bit MCU with more RAM space, which is very suitable for running real-time feature extraction on chip. The BLE is used because the Android 4.4.2 smartphone has embedded BLE microprocessors with capabilities for connecting wirelessly to external biomedical sensors by lower power consumption. Generally, wireless transmission is the most power-demanding component for wireless sensor nodes [37]. Therefore, the combination of on-chip feature extraction and BLE in this study aims to reduce the transmitted data and the whole system power consumption.

#### 2.1.3. Signal Analysis and Feature Extraction

A digital band-pass filter (4–30 Hz) is implemented in SPU to further filter EEG signals, particularly the power line noise (60 Hz). For each 2-s EEG and gyroscope epoch, the following feature extraction approaches are operated respectively.

On-chip EEG Feature Extraction: Relative band power (RBP). First, FFT power is calculated as the sum of the squared FFT magnitude of the EEG signals; then, the RBP is calculated by dividing the FFT power of one EEG band by the sum of the FFT power of all three EEG bands, as shown in Equation (1), where, $EEG_{band}^{i}$ = { θ, α, β }.

(1) $$RBP(EEG_{band}^{i})=\frac{Power(EEG_{band}^{i})}{\sum_{i=1}^{3}Power(EEG_{band}^{i})}\times 100\%$$

On-chip Gyroscope Feature Extraction: Movement power (MP). The MP is developed for accelerometer by Da-Wei Chang *et al.* [38]. Very similar to their work, the MP is applied to gyroscope analysis in this study. First, the gyroscope magnitudes of the three axes are averaged. Then, the standard deviation of the averaged gyroscope signals in one epoch, defined as the MP, is calculated as shown in Equation (2).

(2) $$\begin{array}{c} Gyro_{avg}=(Gyro_{x}+Gyro_{y}+Gyro_{z})/3\\ MP=STD(Gyro_{avg}) \end{array}$$

Therefore, a set of four features (Per (θ), Per (α), Per (β) and MP) were extracted from each 2-s epoch. In order to synchronize the extracted features with the 1-min video-based ground truth (See Section 3) every 30 sets of feature were successively averaged before applying each SVM operation.

### 2.2. Classifier

**Figure 3:**
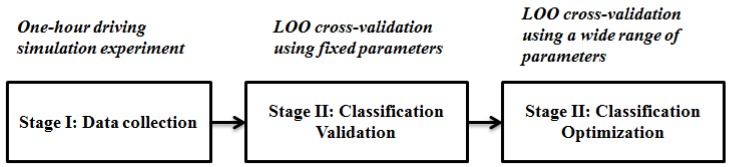
The classification model building chain containing three different tasks: data collection, classification validation and classification optimization.

To test the classification performance with the above-mentioned feature set, a SVM binary classifier is used. As shown in Figure 3, applying this method includes three stages—Stage I: Data collection, Stage II: Validation and Stage III: Optimization. Stage I involves the one-hour simulated driving experiment (See Section 3). Stage II and Stage III involve the leave-one-out (LOO) cross-validation and its optimization respectively. The following subsections briefly introduce the SVM theory and introduce in detail the Stage II and Stage III. All the classifier implementation procedures are done using the highly successful LibSVM [39].

#### 2.2.1. Theoretical Principle of SVM Classifier

A binary SVM classifier outputs a class label (e.g., +1: slightly drowsy events or −1: alert events) for each input dataset comprising several features based on the sign function $f(\vec{x})=sign(\vec{w}\cdot \Phi (\vec{x})+b).$ The $\vec{w}$ is a vector perpendicular to the decision surface and b is a scalar (decision surface bias). $\Phi (\vec{x})$ is the mapping function which maps each input dataset $\vec{x}$ from the input space ${\text{ℜ}}^{n}$ to a higher dimensional feature space H by using kernel functions (e.g., radial basis function (RBF)). The kernel function can be denoted as $K(\vec{x},{\vec{x}}^{\prime })=\Phi (\vec{x})\cdot \Phi ({\vec{x}}^{\prime })$, where $\vec{x}$ and ${\vec{x}}^{\prime }$ denote the inputs in original space (${\text{ℜ}}^{n}$) and nonlinear space (H), respectively; then the linear kernel and RBF kernel are defined by following equations:

1) Linear kernel
  (3) $$K(\vec{x},{\vec{x}}^{\prime })=\vec{x}\cdot {\vec{x}}^{\prime }$$
2) RBF kernel
  (4) $$K(\vec{x},{\vec{x}}^{\prime })=\exp(-\frac{{\left\|\vec{x}-{\vec{x}}^{\prime }\right\|}^{2}}{2g^{2}})$$

Here, parameter *g*, supplied by the user, acts as the scaling factor or radius of RBF kernel. Small value of *g* produces smooth decision boundaries, preventing overfitting of the model to the data samples; while high value of *g* generates complex decision boundaries which has the possibility to be too specific to the data samples, resulting overfitting of the model.

**Figure 4:**
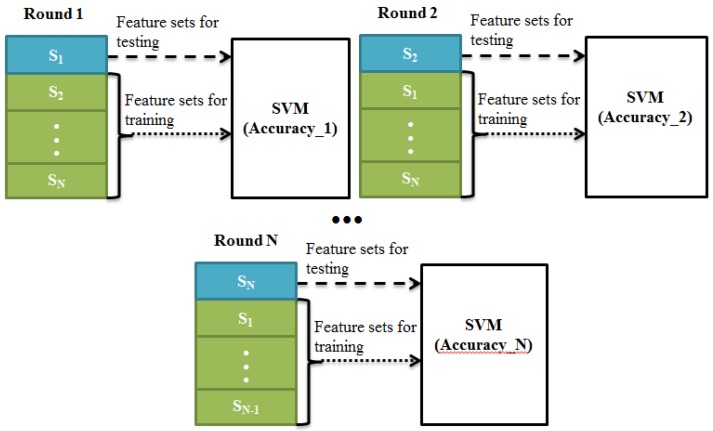
The procedure of LOO cross-validation and optimization, where S_i_ means the *i*-th subject and Accuracy_i means the classification accuracy for *i*-th Round.

#### 2.2.2. LOO Cross-Validation and Optimization

The procedure of LOO cross-validation and optimization is depicted in Figure 4. For evaluating classification performance, the classification accuracy which is based on LOO cross-validation for all participants was calculated. The specific steps are as follows: (1) Omit a subject from all available feature sets; (2) Train the classifier; (3) Test the omitted feature sets; (4) Repeat the steps that are listed above until each subject has been omitted and tested once; (5) Calculate the average classification accuracy (e.g., based on the N Rounds). In order to get the optimized average accuracy, the regulation parameter *C* and value of *g* will be updated from 0.01 to 10 in steps of 0.01 and each updating step corresponds to each cycle of Round 1 to N.

### 2.3. Smartphone

Since the initial release of iPhone and Android phones in 2007 and 2008, respectively, there has been an unprecedented increase in the number of smartphone subscribers in the world. Not surprisingly, smartphones have been proposed as an inexpensive platform for data acquisition and processing.

**Figure 5:**
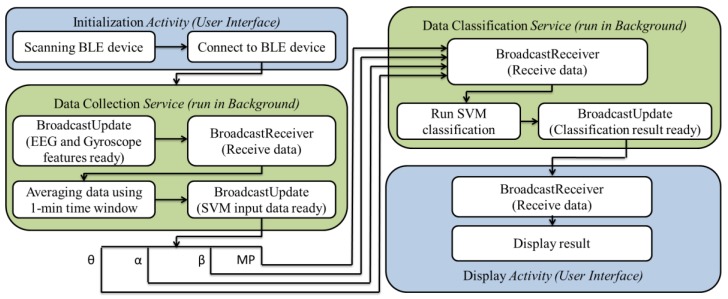
The working flowchart of smartphone which consists of two *Activities* (User interfaces) and *Services* (The functions running in background).

In this study, an Android smartphone serves an on-line Data-to-Knowledge platform. In Android OS, each *Activity* corresponds to each user interface. Each user interface has a short lifetime due to the power save mode of smartphone screen; while *Service* is independent of *Activity* and specifically designed to run the repeating task in background. So, in order to achieve low-power purpose, we employed *Service* to collect data continuously in background instead of keeping screen ON. It is also important to note that we employed another *Service* to classify the collected data continuously (as shown in Figure 5). The two *Services* mainly involved two Android API components: *BroadcastUpdate* and *BroadcastReceiver*. By using the two components, the smartphone just needs to burst out the collected data when data is ready and then powering back down again. This not only can reduce power consumption, but also can release the workload of core processor.

The SVM binary classification model is embedded in the smartphone. This model was well trained on a PC first using MATLAB version R2012a. After that, the parameters of the well-trained SVM model were hardcoded in text-file format and stored on the Secure Digital (SD) card memory of the smartphone. Meanwhile, the LibSVM library was embedded into the smartphone. Finally, the smartphone can call library functions such as svm.svm_load_model and svm.svm_predict to run SVM in real-time.

## 3. System Evaluation Design and Materials

The feasibility of the proposed DDD system is tested in four aspects. First, we test the EEG signal quality from the dry electrodes used. Then, before applying to SVM classifier, the extracted EEG and gyroscope features from a simulated driving experiment will be analyzed and compared between alert and slightly drowsy group using ROC_area_. ROC_area_ is the area under the receiver operating characteristic (ROC) curve and reference line (as shown in Figure 5c), which is an effective measure of the class-discrimination capability of a specific feature [13,40]. Its value can be any value from 0.5 to 1. A value of 0.5 implies that the features are completely overlapped and thus non-separable; while a value of 1 implies that the features are completely separable. The ROC analysis was implemented on IBM SPSS Statistics software (IBM, Armonk, NY, USA), where the features need to be analyzed (“Test variable” in SPSS) are Per(θ), Per(β) and Per(α). The “State variable” is ground-truth-based label (alert and slightly drowsy). Next, the detection accuracy of the proposed system will be evaluated. Finally, the system real-time performance including the system computational cost and power consumption will be investigated. The following part introduces in detail the simulated driving experiment.

According to our previous experiences [12,13], one-hour monotonous driving after lunch (typically around 1:00–2:30 pm) results in drowsiness in a majority of subjects. Therefore, to evaluate the proposed system, six subjects, possessing valid driver’s licenses, participated in the one-hour monotonous driving simulation experiment. The driving simulation environment consisted of a commercial truck driving simulator (Euro Truck Simulator 2), a Logitech^®^ steering wheel, acceleration and brake pedals. During experiment, the participants drove the virtual truck with a few road simulators on the highways and experienced various realistic cognitive loads, such as keeping or changing lanes to avoid collisions, turning on windshield wiper when driving in rainy conditions, and early deceleration before red lights. On the day of the experiment, subjects were prohibited from drinking tea or consuming anything containing caffeine. Moreover, consumption of soporific medicines, such as standard cold medications, was prohibited. Before the experiment, each subject was given ten minutes to familiarize himself/herself with the operation of the driving simulator. For labeling the true alert and slightly drowsy events, Wierwille scale was used. Wierwille scale is a widely-used video-based DDD ground truth [23], which can classify the driving status into slightly drowsy driving according to following indicators: (1) increase in duration of eye blinks; (2) possible increase in rate of eye blinks; (3) increase in duration and frequency of sideway glances; (4) appearance of “glazed-eye” look; (5) appearance of abrupt irregular movements—rubbing face/eyes, moving restlessly on the chair; (6) Abnormally large body movements following drowsiness episodes; (7) occasional yawning. We can see clearly that indicator (5)–(7) can directly cause head-movements and thus can be easily captured by gyroscope sensor.

## 4. System Evaluation Results

### 4.1. EEG Signal Quality Test

**Figure 6:**
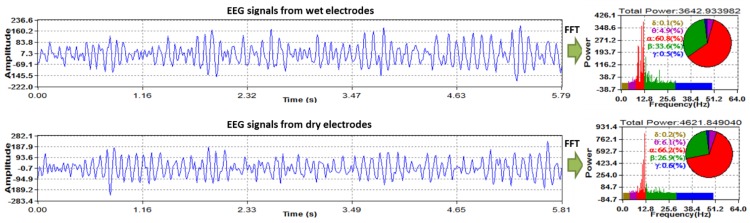
Comparison of EEG signals (line chart) and band power (pie chart) between wet electrodes (the top) and dry electrodes (the bottom). For the line chart, *X*-axis indicates the time (second). *Y*-axis indicates the amplitude of the digitalized EEG samples which are already filtered by the digital band-pass filter (4–30 Hz) in SPU. For the pie chart, the *X*-axis indicates the frequency ranged from 0–64 Hz (half of the sampling rate 128 Hz). *Y*-axis indicates the magnitude of FFT power.

The test of EEG signal quality can be divided into two separate phases: (1) Getting EEG signals from dry electrodes; (2) Getting EEG signals from wet electrodes. Both phases were based on the same SIU and SPU and the same positions (O1 & O2). One subject was instructed to close his eyes for five seconds. What we expected to be common between these two sessions was the appearance of obvious α rhythmicity in time domain and the largest power percentage for α band in frequency domain. What exactly we observed is shown in Figure 6, which is just consistent with our expectation.

### 4.2. Feature Analysis

In total, 266 labeled feature sets containing 68 alert feature sets and 198 slightly drowsy feature sets were collected through this one-hour driving experiment from six subjects. The labeled drowsy events also included moderately drowsy events, significantly events and extremely drowsy events (a total of 94 events). However, the focus of this study is to build a binary classification model (alert events *vs.* slightly drowsy events) with an aim to enable the early-detection of driver drowsiness, so the analysis of mid- or late-stage drowsiness analysis are excluded in this study.

**Figure 7:**
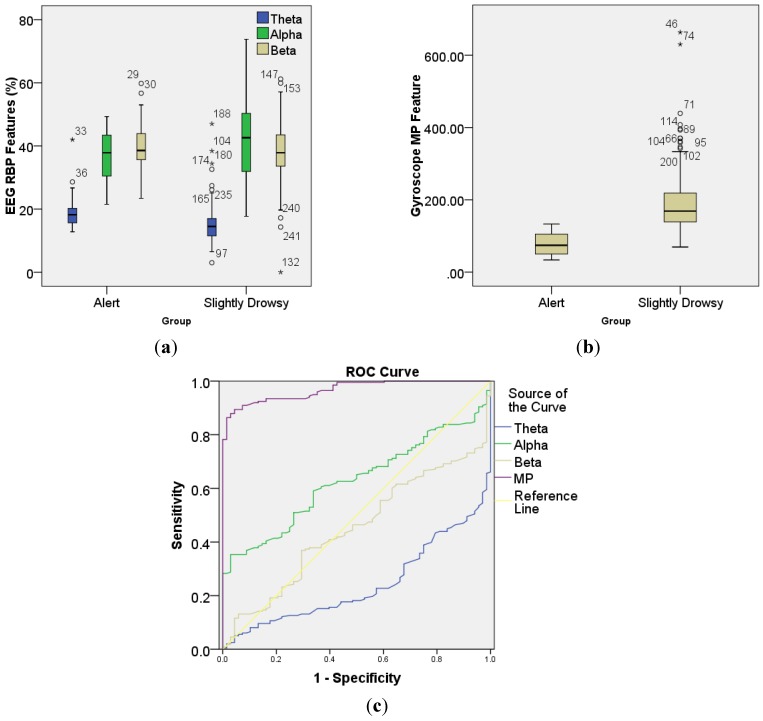
Box-Whiskers plots of (**a**) EEG and (**b**) gyroscope features. The boxes have three lines to present the values for first quartile (the bottom), median, and third quartile (the top) for column data. The length between the first quartile (Q1) and the third quartile (Q3) is called interquartile range (IQR). Two addition lines at both ends of the whisker indicate the Q1 − 1.5 × IQR and Q3 + 1.5 × IQR value of a column data. Any data not included between the whiskers is plotted as outliers represented by “o” for mild outliers and “*” for extreme outliers. The number next to the outlier is the number of the data in that column, called case number; (**c**) ROC curve showing sensitivity (possibility of true drowsy event) and 1-specificity (possibility of false drowsy event) for extracted EEG and gyroscope features.

**Figure 8:**
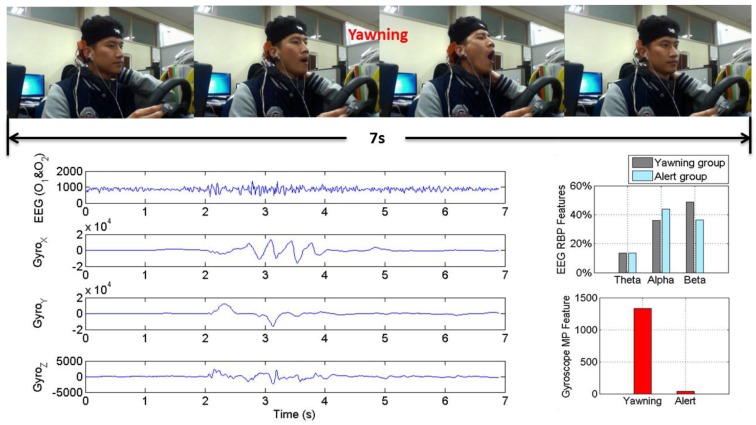
The typical slightly drowsy symptom, yawning, captured by video as well as EEG and gyroscope from a representative subject. The blue line charts represent EEG raw signals, *X*-axis, *Y*-axis and *Z*-axis signal of the gyroscope, respectively. The two bars on the right side of the line charts represent the EEG RBP features (the top) and the gyroscope MP features (the bottom).

Each feature set consists of four extracted features: RBP (θ), RBP (α), RBP (β) and MP. The Box-Whiskers plots (Figure 7a,b) show that the median values for EEG features RBP (θ), RBP (α), RBP (β) and Gyroscope feature MP are 18.2%, 37.9%, 38.6% and 74.1 for alert group and 14.5%, 42.6%, 37.9% and 168.7 for slightly drowsy group. The trend of EEG features is consistent with previous EEG conclusion [12,17,19], that is, when a driver passes from the alert to the drowsy stage, β power decreases and α power increases and becomes abundant. However, ROC analysis (Figure 7c) shows that the EEG features are not clearly separable with ROC_area_ = 0.760 for RBP (θ), ROC_area_ = 0.628 for RBP (α) and ROC_area_ = 0.550 for RBP (β); while gyroscope feature MP shows an outstanding class-discrimination capability with ROC_area_ = 0.967. To explain this further, the EEG features and MP features of one representative subject that were extracted from yawing movement (the most frequent slightly drowsy symptom during driving experiment if compared to rubbing eyes movements and moving restlessly movements) are shown in Figure 8, where we can see clearly that the MP has a significant increase when this subject was yawning, while his α power decreased and β power increased during this period, which is just contrary to previous conclusions. This phenomenon is expected since the intermittent head-movements that are caused by slightly drowsy symptoms lead to poor contact between the dry electrodes and scalp. This results in artifacts and friction noise for EEG signals [41], but it is useful contextual information for DDD that can be easily captured by the gyroscope sensor.

### 4.3. Detection Accuracy

Letting negative label “−1” and positive label “+1” represent alert class and slightly drowsy class respectively, the LOO binary classification using EEG features alone, MP alone and the hybrid features was done. The results are summarized in Table 1, where Accuracy (Acc), sensitivity (Sens), and specificity (Spec) are calculated as shown in Equation 5, where TP is true positive, TN is true negative, FP is false positive, and FN is false negative. Therefore the Sens means how well this classifier can recognize the driver who is in the slightly drowsy status, while the Spec means how well this classifier can recognize the driver who is in alert status.

(5) $$\begin{array}{l} Accuracy=\frac{TP+TN}{TP+TN+FP+FN}\\ Sensitivity=\frac{TP}{TP+FN}\\ Specificity=\frac{TN}{TN+FP} \end{array}$$

Each Acc value in Table 1 has been optimized by using a search procedure with *C* and *g* = {0.01 ~ 10} in steps of 0.01. It is important to note that the performance of Linear-SVM using hybrid feature was better than RBF-SVM; while the performance of RBF-SVM using EEG feature alone and MP feature alone was better than Linear-SVM. The best Acc for EEG features alone was 82.71% using RBF kernel of *C* = 2 and *g* = 0.1. The best Acc for MP feature alone was 92.86% using RBF kernel of *C* = 1 and *g* = 0.01. It is also important to observe that using EEG feature alone obtained a very low Spec (0% in Linear kernel and 45.59% in RBF kernel) indicating many alert events were misclassified to slightly drowsy events. However, the result of using hybrid features enables in better differentiation between alert events and slightly events (Acc = 96.24%, Sens = 96.46%, Spec = 95.59%) by providing better contextual information. This indicates the advantage of the proposed approach.

**Table 1:** Results of the leave-one-out (LOO) cross-validation experiments using EEG feature sets alone, gyroscope feature alone and the hybrid feature.

**Kernel**	**EEG Features (RBP (θ), RBP (α), RBP (β))**			**Gyroscope Feature MP**			**Hybrid Features (RBP (θ), RBP (α), RBP (β), MP)**		
	Sens	Spec	Acc	Sens	Spec	Acc	Sens	Spec	Acc
Linear	100	0	74.43	96.46	63.24	87.96	96.46	95.59	96.24
	*C* = 0.01			*C* = 0.01			*C* = 2		
RBF	95.45	45.59	82.71	93.43	91.18	92.86	96.46	91.18	95.11
	*C* = 2			*C* = 1			*C* = 5		
	*g* = 0.1			*g* = 0.01			*g* = 0.01		

### 4.4. Real-Time Performance

**Figure 9:**
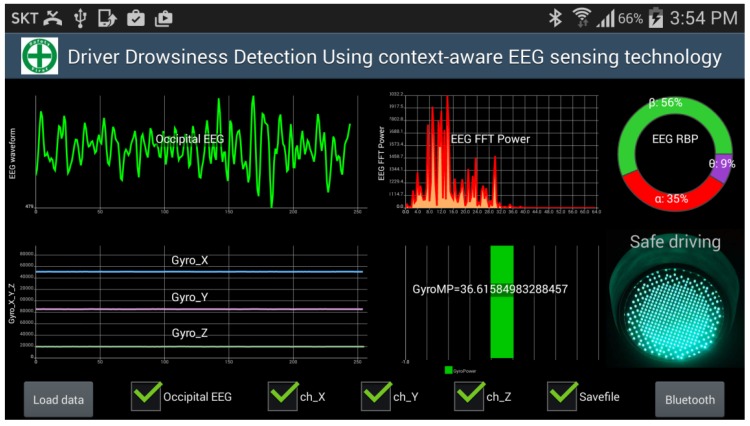
Screenshot of the Android smartphone application that shows the EEG and gyroscope features, the estimation of the driving status, and the raw data of EEG and 3-axis gyroscope.

Our proposed system includes a 32-bit MCU and a commercial smartphone for on-line feature extraction and classification, respectively. Therefore, for real-life application, the computational cost of the proposed feature extraction approach and drowsiness level prediction model is also investigated. First, we programed the MCU to print out the system time before and after running FFT via serial port terminal. We found that the time difference can be ignorable at millisecond level. Similarly, for the smartphone, we used the debugging tool *Logcat* in the Android developing SDK (Android Studio beta ver 0.8.6) and let *Logcat* print out the system time before and after running SVM and 4-ms time delay is found. Also, as can be seen in Table 2, the power consumption and estimated battery life of the developed headset for remote feature extraction (where both EEG and gyroscope feature extraction are performed remotely (in smartphone)) is compared to that with local processing (where both EEG and gyroscope feature extraction is performed directly on the MCU chip of headset). In addition, the power consumption using BLE module is compared to that using conventional Bluetooth v2.0 EDR+ module [42]. We can see clearly that five-hour and three-hour battery life was extended for BLE and Bluetooth v2.0 EDR+ based system, respectively, by using local processing method. To demonstrate the mobile platform for on-line analysis, the screenshot of the smartphone application which is using remote feature extraction is shown in Figure 9.

**Table 2:** Results of the headset battery life comparison of remote and local feature extraction.

Condition		Feature Extraction Approach	Power Consumption (mA)	Battery Life (h)
Power supply 3.6 V	BLE	Remote	63	41
Battery capacity: 2600 mA·h		On chip	56	46
Sampling rate: 128 Hz	Bluetooth v2.0 EDR+	Remote	82	32
ADC resolution: 12 bits		On chip	75	35
Bluetooth (slave) : Active				
Baud-rate: 115,200 bps				

## 5. Discussion

### 5.1. Principle Results

A new approach for classification of driver drowsiness is proposed in this study. We use gyroscope to measure the intensity of the head movement and detect driver drowsiness with EEG signal together. Gyroscope features are used and compared with FFT-based EEG features. According to previous studies, it is clear that FFT-based features are effective in detecting driver drowsiness from EEG. However, in this study, when it comes to the stage of classification of early drowsiness, the information provided by EEG alone is proved to be insufficient. The information presented in the head movements (early drowsy symptoms) is lost. When taking gyroscope into consideration, it provides richer contextual information which is very useful in detecting early-stage drowsiness. This rich information representation is the reason for better detection accuracy of 96.24% using gyroscope and EEG features together against the accuracy of 82.71% using EEG alone and 92.86% using the gyroscope feature alone in this study.

### 5.2. Comparison with Prior Work

#### 5.2.1. EEG *versus* Other Physiological Signals

Heart rate variability (HRV) is the widely-used drowsiness indicator for ECG or Photoplethysmogram signals [13]. However, the minimum and regular time window for FFT-based HRV analysis is three minutes and five minutes, respectively [43], which is longer than the 1-min length of time window used in our study. Clearly, from the point view of timeliness, EEG signal is more suitable for DDD application as its detection result could be delivered to drivers as early as possible. The physiological reasoning behind the shorter time window for EEG analysis should be its direct relationship to drowsiness. Even if more advanced pattern recognition techniques were used, the performance using EEG alone is still better than using other physiological signal alone (e.g., ECG, EOG) as long as under the same conditions (e.g., the same subjects, ground truth and experimental environment) [18].

#### 5.2.2. Signal Processing Comparison

To compare different techniques of EEG processing for drowsiness detection, three more recent research papers are used here. Unlike the FFT-based RBP features and SVM classifier used in our study, Garcés *et al.* [44] obtained accuracy of 83.6% for drowsiness detection and 87.4% for alertness detection by using artificial neural network and a wide range of EEG features including time-domain features (e.g., minimum, maximum and standard deviation (STD) of EEG amplitude), frequency-domain features (e.g., FFT-based central frequency, peak frequency and maximum frequency) and time-frequency-domain features (e.g., Wavelet-based zero-crossing rate and integrated EEG from each of the bands (θ to β)). A major drawback of this study is that they directly categorized driver drowsiness as the sleep Stage I when feedback might be already late. To detect drowsiness earlier, Melia *et al.* [45] conducted a study based on the MSLT and WMT standard as introduced in Section 1. They proposed two novel non-linear EEG features: auto-mutual information function and cross-mutual information function based features. Experimental results show that these non-linear features (ROC_area_ > 0.75) outperform traditional linear features (ROC_area_ < 0.75). However, one limitation for this study is that the subjects used were excessive daytime sleepiness patients instead of normal healthy persons indicating this method is not yet readily applicable to the general population of drivers. Similarly, Chen *et al.* [46] also used nonlinear features to detect drowsiness; however, they used long-term mental calculation to induce drowsiness rather than using a real-life environment (e.g., monotonous driving environment used in our study).

#### 5.2.3. Detection Accuracy Comparison

To compare the detection accuracy with prior work, three EEG-based DDD studies that use the same ground truth Wierwille scale and also combine contextual information are carefully found from the numeric DDD studies. One of them recorded contextual information using motion sensor; while the remaining two studies recorded contextual information using physiological signals. Specifically, Pritchett *et al.* [47] also proposed an EEG-based context-aware solution for DDD. The main difference between the current system and that presented in [47] is that they recorded contextual information from the driver’s seat where a piezoelectric film sensor is attached rather than from the headset directly. In addition, unlike the SVM model used in our study, they used linear regression model to estimate the drowsiness level (dependent variable), in which a wide range of *α* burst parameters and body movement parameters were used as features (independent variables). Experimental results show that hybrid features (*R*^2^ = 0.308) outperform EEG features (*R*^2^ = 0.272), where *R*^2^ is the squared correlation coefficient, a commonly used method of estimating the performance of the proposed regression model [48]. If *R*^2^ is high (maximum value is 1), it can be claimed that the driver drowsiness level and the extracted features have a strong linear relationship and that the performance of the regression model is superior if compared to a low *R*^2^. Clearly, the performance of the regression model above is poor. This is expected because regression models are good for estimating a continuous variable not the discrete labels here (5-level driver drowsiness). Khushaba *et al.* [18] extracted features using normalized logarithmic energy of the wavelet-packet coefficients from 5-channel physiological signals: 3-ch EEG, 1-ch ECG and 1-ch EOG. Then, a fuzzy mutual information based method is used to select features. Finally, the kernel spectral regression based linear discrimination analysis model obtains an outstanding detection accuracy of 97% for 5-level classification including mid- or late-stage drowsiness classification. However, they did not mention the detection accuracy between alert events and slightly drowsy events which indicates the capability to early detect driver drowsiness. Also, Khushaba *et al.* [49] extracted time domain autoregressive features from the aforementioned 5-ch signals and then uncorrelated fuzzy neighborhood preserving analysis is used to select features. Finally, they can detect alert events and slightly drowsy events with 94% accuracy using RBF-SVM classifier. These pattern recognition techniques used above are more complicated than that used in the proposed method. However, they do not outperform our proposed method (94% *vs.* 96.24%). This result shows that the selection of signal source is still the most important part of designing the best detection models. In addition, compared with the two studies, this study has several advantages. First, we used dry electrodes instead of conventional wet electrodes, which show more realistic detection accuracy. Second, we used 4-ch signals (1-ch EEG + 3-ch gyroscope) instead of 5-ch physiological signals (3-ch EEG + 1-ch ECG + 1-ch EOG), which is less intrusive. Third, our proposed approach is evaluated on miniaturized and source-limited devices instead of laboratory-oriented devices, which show strong practical utility.

### 5.3. Limitation

This paper has been primarily focused on the design and implementation of an EEG headset with integrated gyroscope sensor with an aim to enable the early-detection of driver drowsiness. There are no arousing feedback methods (thus drivers’ attention cannot be boosted) in this study. However, this can be easily overcome by setting a warning signal, such as an auditory tone-burst [21,50,51].

## 6. Conclusions and Future Work

The design and evaluation of a context-aware EEG headset system is described in this paper. The system uses a Bluetooth-enabled, EEG and gyroscope sensor-equipped headset and a machine learning model-enabled smartphone aimed to detect driver drowsiness at its early stage. This not only shifts DDD from being a reactive to a preventive driver safety technology, but also achieves a simple and inexpensive on-line analysis platform. Further studies considering an effective brainwave entrainment technology need to be performed in order to develop a real-time driver alertness boosting method. In addition, we are considering enhancing the wearability of the developed headset using more flexible 3D printer material. Also, an extensive field test needs to be established before applying the proposed system to a practical environment.
